# Glutamate Carboxypeptidase II Inhibition Behaviorally and Physiologically Improves Pyridoxine-Induced Neuropathy in Rats

**DOI:** 10.1371/journal.pone.0102936

**Published:** 2014-09-25

**Authors:** Michelle C. Potter, Krystyna M. Wozniak, Noelle Callizot, Barbara S. Slusher

**Affiliations:** 1 Brain Science Institute, Johns Hopkins University School of Medicine, Baltimore, Maryland, United States of America; 2 Department of Neurology, Johns Hopkins University School of Medicine, Baltimore, Maryland, United States of America; 3 Department of Psychiatry, Johns Hopkins University School of Medicine, Baltimore, Maryland, United States of America; 4 Department of Neuroscience, Johns Hopkins University School of Medicine, Baltimore, Maryland, United States of America; 5 Department of Neuropharmacology, Neuro/Sys, Gardanne, France; “Mario Negri” Institute for Pharmacological Research, Italy

## Abstract

Pyridoxine is used as a supplement for treating conditions such as vitamin deficiency as well as neurological disorders such as depression, epilepsy and autism. A significant neurologic complication of pyridoxine therapy is peripheral neuropathy thought to be a result of long-term and high dose usage. Although pyridoxine-induced neuropathy is transient and can remit after its withdrawal, the process of complete recovery can be slow. Glutamate carboxypeptidase II (GCP II) inhibition has been shown to improve symptoms of both chemotherapy- and diabetic-induced neuropathy. This study evaluated if GCP II inhibition could behaviorally and physiologically improve pyridoxine-induced neuropathy. In the current study, high doses of pyridoxine (400 mg/kg, twice a day for seven days) were used to induce neuropathy in rats. An orally bioavailable GCP II inhibitor, 2-(3-mercaptopropyl) pentanedioic acid (2-MPPA), was administered daily at a dose of 30 mg/kg starting from the onset of pyridoxine injections. Body weight, motor coordination, heat sensitivity, electromyographical (EMG) parameters and nerve morphological features were monitored. The results show beneficial effects of GCP II inhibition including normalization of hot plate reaction time, foot fault improvements and increased open field distance travelled. H wave frequency, amplitude and latency as well as sensory nerve conduction velocity (SNCV) were also significantly improved by 2-MPPA. Lastly, GCP II inhibition resulted in morphological protection in the spinal cord and sensory fibers in the lumbar region dorsal root ganglia (DRG). In conclusion, inhibition of GCP II may be beneficial against the peripheral sensory neuropathy caused by pyridoxine.

## Introduction

Glutamate carboxypeptidase II (GCP II; also known as N-acetylaspartyglutamate (NAAG) peptidase) is a membrane-bound metalloenzyme that cleaves the abundant neuropeptide NAAG to N-acetylaspartate (NAA) and glutamate [1]. NAAG is one of the most widespread peptide transmitters in the brain and is a type 3 metabotropic glutamate receptor (mGluR3) agonist [2], [3]. GCP II inhibitors have been shown to increase extracellular NAAG, decrease glutamate and prevent neurotoxicity in several preclinical disease models where excess glutamatergic transmission is presumed pathogenic [4]. These include pain [5], [6], [7], [8], brain ischemia/stroke [1], motoneuron disease [9], brain and spinal cord injury [10], [11], peripheral neuropathy [12], [13], epilepsy/seizures [14] and drug abuse [15], [16]. The specific GCPII inhibitor used in this current study, 2-(3-mercaptopropyl) pentanedioic acid (2-MPPA), also known as GPI5693, is the first orally bioavailable GCPII inhibitor described [17]. 2-MPPA has also been administered to human volunteers and was well tolerated with no reports of adverse CNS effects [18]. In previously published studies 2-MPPA, at similar or greater doses to that tested here, have been shown not to cause any effect when given alone to rats or mice [19], [20], [21], [22].

Pyridoxine is an essential water soluble vitamin (B_6_) that is an important coenzyme in many biochemical reactions in the body [23], [24]. However, large doses of pyridoxine have been shown to induce peripheral neuropathy affecting large sensory fibers of the dorsal root ganglion (DRG) with severe loss of proprioceptive function in patients [23], [24], [25]. Similar findings have also been extensively reported in animal models [26], [27]. The primary site of injury is the cell body of DRG neurons which in turn leads to damage of the integrity of their long myelinated fibers and ultimately to cell death. Vacuolization, increased dense bodies, neurofilament aggregates and chromatolysis have been reported in the soma of affected cells [28], [29]. Decreased large caliber axons and argyrophilic axonal neurodegenerative profiles in the dorsal columns have also been described [28], [29]. Though the exact mechanism as to how pyridoxine is leading to neurodegeneration is unknown, several hypotheses have been proposed such as the negative impact on other B vitamins [30], [31], competitive inhibition of pyridoxol phosphate, the formation of reactive quinine methide, and the interruption of local chelation of magnesium [29], [32]. The susceptibility of neurons in the peripheral nervous system is likely due to a less complete blood-nerve barrier compared to the blood-brain barrier that protects the brain from high levels of circulating pyridoxine [29], [32]. Regardless of the precise mechanism, chronic administration of 400 mg/kg pyridoxine twice daily to rats reliably induces profound proprioceptive loss similar to that observed in humans [26], [27] and thus has become an established preclinical model of sensory neuropathy. The neurodegeneration seen with this model is similar to that observed in clinical diabetic neuropathy [29].

The current study was designed to evaluate the potential neuroprotective effect of a GCP II inhibitor in a model of pyridoxine-induced peripheral neuropathy. We report that daily administration of the potent orally available GCP II inhibitor, 2-MPPA, protects against loss in both motor and sensory function as well as neurodegeneration induced by pyridoxine.

## Materials and Methods

### Animals

Thirty young adult female Sprague Dawley rats (180–230 g) were obtained from Charles River Laboratories (France), group-housed (2 per cage) and maintained in a well-ventilated vivarium with controlled temperature (21–22°C), a reversed light-dark cycle (12 h/12 h) and free access to food and water. All animal procedures were carried out in strict accordance with the recommendations of the French Ministry of Agriculture. The protocol was approved by Anses’s Committee for Ethical Standards.

### Pyridoxine-induced neuropathy model

400 mg/kg pyridoxine (Sigma, Saint-Quentin-Fallavier, France) or vehicle was injected intraperitoneally into each rat, twice daily (morning between 7–8 am and afternoon between 4–5 pm) for 7 days as previously described [26], [27]. 400 mg/kg is equivalent to approximately 57 mg/kg in clinical dosing when converted to an equivalent surface area dose in humans. The current tolerable upper intake of vitamin B6 is around 100 mg per day in humans in the US [33]. The pyridoxine was formulated at 50 mg/ml in sterile 0.9% sodium chloride, prepared immediately before injection.

### Drug administration

**Figure 1:**
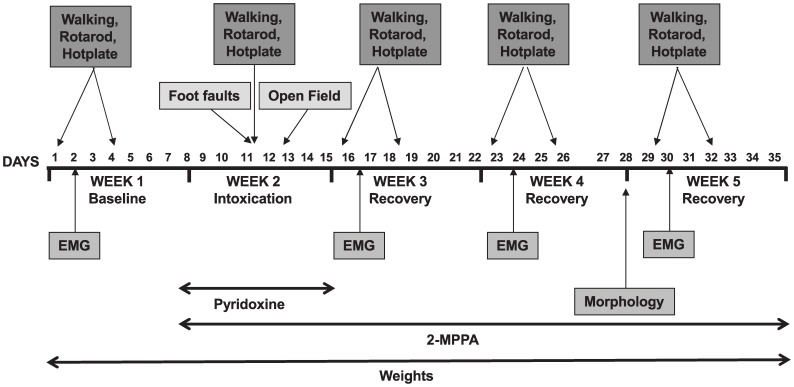
Timeline of experiment.

Three groups (N = 10 rats/group) were used for the study: (a) non-intoxicated control group receiving vehicle; (b) pyridoxine-intoxicated group receiving vehicle and (c) pyridoxine-intoxicated group receiving the GCP II inhibitor, 2-MPPA, at a concentration of 30 mg/kg administered via oral gavage (the solution was prepared every other day in a vehicle of 50 mmol/l Hepes-buffered saline, adjusted to pH 7 and stored at 4°C). 2-MPPA and vehicle were dosed daily (morning between 9–10 am) from the first day of pyridoxine intoxication until the end of the entire study. This study was performed over 5 weeks with various parameters being measured during each week as detailed below and in Figure 1. 2-MPPA was administered 1 hour prior to behavior testing for all tests.

### Walking test

This test was conducted using a rod of 5.5 cm diameter and 100 cm length, maintained horizontally 40 cm above a table. The rod was graduated to allow for the measurement of the animal walking distance. Three trials per session were performed. For each trial (60 s maximum), each rat was placed at one extremity of the rod and the time needed to walk the 1 m distance was recorded. Should the animal fall down or be unable to walk the 1 m distance, 60 s were credited. For each animal, the mean duration of the 3 trials was calculated.

### Rotarod test

An animal was placed on a 5 cm diameter automated rotarod (Bioseb, Paris, France) using settings of 12 rotations per min. The rat was placed on the rotating rod, and the time it remained on the rod was recorded (180 s maximum). If the animal fell off the rod before 180 s, an additional trial was performed (3 trials maximum). The mean duration of the successive trials was calculated.

### Hot plate

Rats were placed in a glass cylinder of 17 cm height and 21 cm diameter on a hotplate (Bioseb, France) set to 55°C and the amount of time it took for the rat to lift it’s paw (reaction time) was recorded.

### Foot fault counting test

Rats were tested for placement dysfunction of limbs using a modified foot fault test [34]. The apparatus consists of a grid floor that is raised above a surface. The rat is placed at one end of the grid and monitored as it maneuvers across the grid as previously described [34]. With each weight bearing step the paw may fall or slip between the grids. The number of foot placement errors as the animal walks across the grid is recorded. An error is counted whenever a foot misses a grid bar and extends downward between the bars. For this experiment, each rat was put on the grids on the fourth day of pyridoxine injections and its behaviors on the grid within three minutes were recorded.

### Open field

Rats were put into the individual open field boxes (Columbus Instrument, Ohio) and their spontaneous locomotor behavior was automatically recorded. The total distance travelled within 60 minutes was recorded.

### Electrophysiological recordings

EMG recordings were performed using a Neuromatic 2000 M electromyograph (Dantec, Les Ulis, France). Rats were anaesthetized with IP injection of 60 mg/kg ketamine chloral hydrate (Imalgène 500, Rhône Mérieux, Lyon, France). H wave distal latency and amplitude were recorded in the plantar hindpaw muscle after stimulation of the sciatic nerve. A reference electrode and an active needle were placed in the plantar side of the right hindpaw. A ground needle was inserted into the lower back of the rat. The sciatic nerve was stimulated with a single 0.2 ms pulse at an intensity of 12.8 mA. The amplitude (mV) and the latency (ms) of H waves were measured. SNCV was also recorded. The tail skin electrodes were placed as follows: a reference needle inserted at the base of the tail and an anode needle placed 30 mm away from the reference needle towards the extremity of the tail. A ground needle electrode was inserted between the anode and reference needles. The caudal nerve was stimulated with a series of 20 pulses (for 0.2 ms) at an intensity of 12.8 mA. The response average was calculated.

### Morphology

Morphological analysis was performed on Day 28, 2 weeks after the pyridoxine intoxication (see Figure 1). For review see [27]; briefly, rats were anaesthetized with an IP injection of 60 mg/kg ketamine chloral hydrate (Imalgène 500, Rhône Mérieux, Lyon, France), euthanised and the L4 and L5 DRG (n = 3 per treatment group) and emerging nerves from a given animal were dissected and then fixed overnight with 4% glutaraldehyde (Sigma) in phosphate buffer (pH 7.4). DRG were fixed in 2% osmium tetroxide (Sigma) in phosphate buffer for 2 h and dehydrated in serial alcohol solutions and embedded in Epon (Epikon 812, Carl Roth KG, Karlsruhe, Germany). Embedded tissues were then placed at 70°C for 3 days of polymerization. Transverse sections (of sensory fibers emerging of L4 or L5 DRG) of 6 µm were made with a microtome and stained with 1% of toludine blue (Sigma, St Louis) for 2 min and dehydrated and mounted in Eukitt. For data analysis, 5 sections (2 fields per section) per animal (N = 3) were studied and the total myelinated sensory fibers from each of these fields were counted and compared across groups. Sections were observed using an optical microscope and morphometric analyses were performed with the aid of the Visiolab 2000 software (Biocom, Paris, France).

The spinal cord was dissected and histologically processed with silver stain for detecting the degenerative fibers. 50um lumbar region sections were cut on a sliding microtome. Free-floating sections were processed for visualization of degenerated nerve processes (argyrophillic axonal profiles) with FD NeuroSilver Kit (FD Neurotechnology, Baltimore, MD).

### Statistics

A global analysis of the data was performed using one factor or repeated measures analysis of variance (ANOVA), one and two way ANOVA tests. Either Fisher’s PLSD, Bonferroni’s, Newman Keul’s or Dunnett’s test were used as posthoc tests (Statview or GraphPad Prism). Moreover, a non-parametric test (Kruskal-Wallis test) was used for the data table describing the electromyographical parameters of sensory reflexes. The level of significance was set at p<0.05 in all cases.

## Results

### GCP II inhibition had no effect on weight loss due to pyridoxine treatment

**Figure 2:**
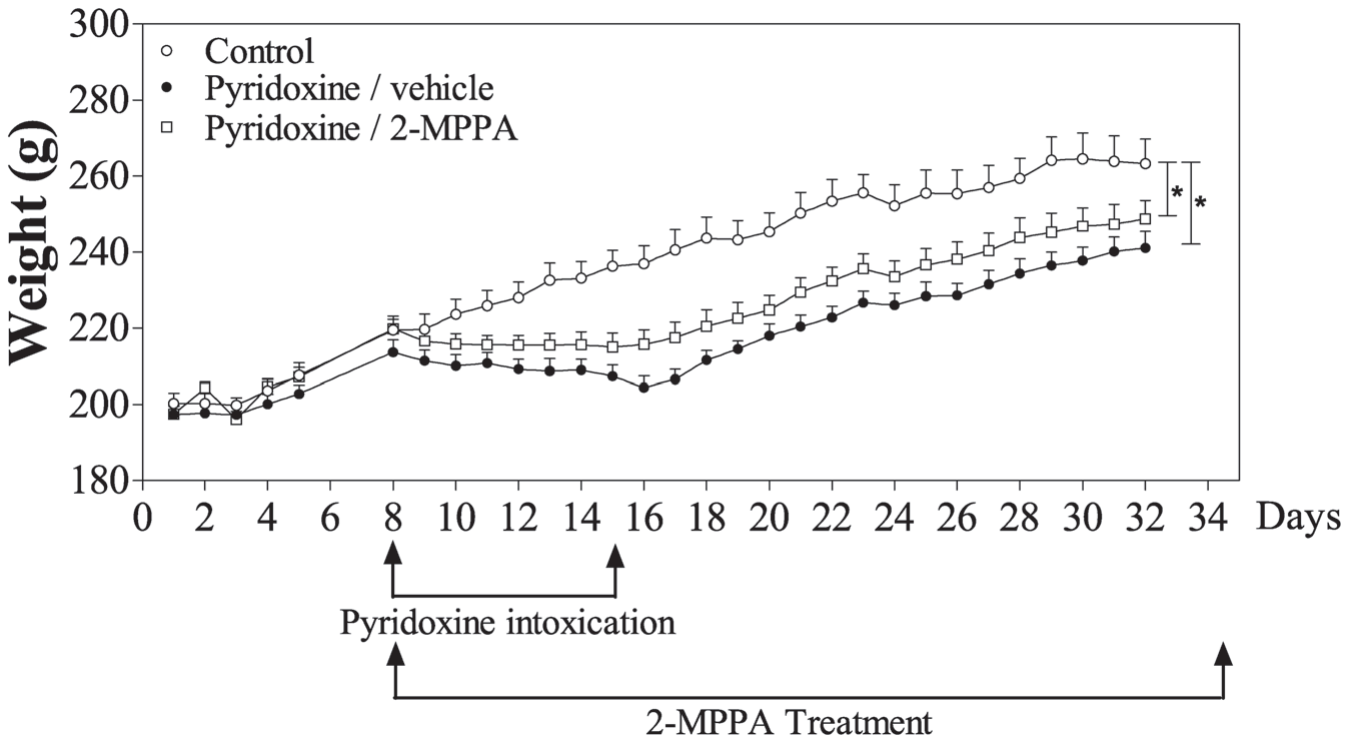
GCP II inhibition did not significantly affect weight loss induced by pyridoxine. Weights were measured every day of the study. A significant difference was observed between the controls and both the pyridoxine treated groups (p<0.05); no difference was observed between the pyridoxine/vehicle and pyridoxine/2-MPPA groups (p>0.05). N = 10/group. Data = mean ± SEM. * = p<0.05.

A significant difference in body weight was observed between the control and pyridoxine treated groups throughout the study [F(2, 24) = 6.89, p<0.05; repeated measures ANOVA] (Figure 2). The GCP II inhibitor treated group showed a less pronounced weight loss during the intoxication and recovery period compared to the vehicle-treated pyridoxine animals, although it was not statistically significant.

### Locomotor deficiencies were improved by GCP II inhibition

**Figure 3:**
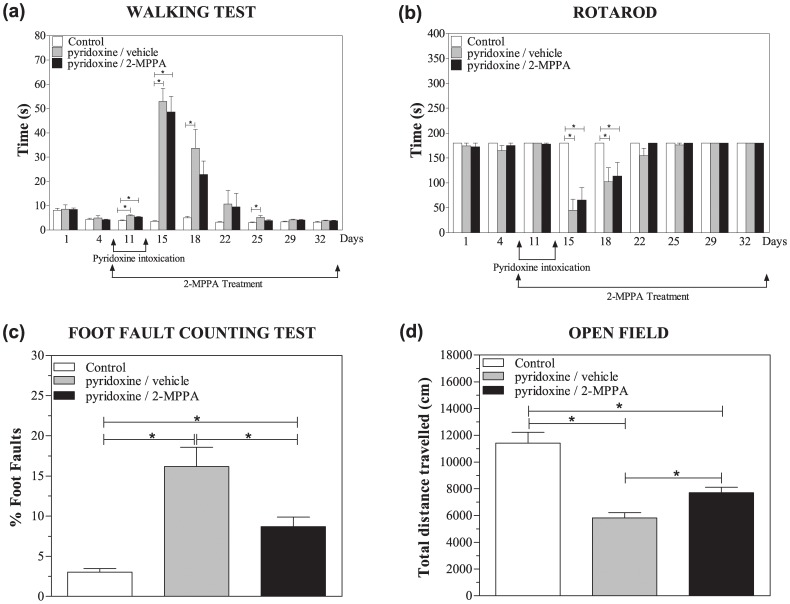
GCP II inhibition improved several pyridoxine-induced locomotor deficits. (a) Walking performance was significantly reduced upon pyridoxine treatment with the control group being significantly different to both pyridoxine treated groups particularly on days 11 and 15. No significant difference was seen between the pyridoxine/vehicle versus pyridoxine/2-MPPA groups on these days. On day 18 and day 25 there was a significant difference between control and pyridoxine/vehicle. However, no difference was observed between the control and the pyridoxine/2-MPPA groups nor between the 2 pyridoxine-treated groups. (b) Pyridoxine intoxication significantly reduced rotarod performance without any benefits of GCP II inhibition. (c) Both pyridoxine groups had significantly increased percentage of foot faults compared to the control group and GCP II inhibition partially blocked this deficit (d). The pyridoxine intoxication significantly decreased distance travelled in an open field and GCP II inhibition partially prevented this decrease. N = 10/group. Data = mean ± SEM. * = p<0.05.

No significant difference in walking time was observed between the 3 groups during the baseline week [F(2, 27) = 0.12, p>0.05, repeated measures ANOVA] (Figure 3a). Comparison of performance during the intoxication and recovery weeks, however, showed a significant difference between the groups [F(2, 24) = 12.82, p<0.05; repeated measures ANOVA] with the control group being significantly different to both pyridoxine treated groups particularly on days 11 and 15 [p<0.05, Fisher’s PLSD]. No significant difference was seen between the pyridoxine/vehicle versus pyridoxine/2-MPPA groups [p>0.05, Fisher’s PLSD]. On day 18 and day 25 there was a significant difference between control and pyridoxine/vehicle (p<0.05; Dunnett’s test) but no difference between the control and the pyridoxine/2-MPPA groups nor between the 2 pyridoxine-treated groups indicating only a trend towards improvement. The walking times decreased throughout the recovery weeks for all the pyridoxine groups and eventually reached the performance levels of the control group.

During testing on the rotarod apparatus during the baseline week there was no difference between groups (Figure 3b). Following pyridoxine treatment, there was a significant difference between treatment groups [F(2, 25) = 8.21, p<0.05; repeated measures ANOVA] with the controls being significantly different to both pyridoxine treated groups. From day 15 to 18, the pyridoxine intoxicated groups were unable to remain on the rotarod, however by day 22 they recovered to normal performance levels. No significant difference was seen between pyridoxine/vehicle versus pyridoxine/2-MPPA [p>0.05, Fisher’s PLSD].

There was a significant difference between the three groups in the percentage of foot faults [F(2, 27) = 17.8; p<0.05; one way ANOVA] on the fourth day of pyridoxine injections (Figure 3c). The pyridoxine/vehicle treated rats had a significantly increased percentage of foot faults compared to the control group (p<0.05; Bonferroni). While there was still a difference between the pyridoxine/2-MPPA group and the control group (p<0.05; Bonferroni) there was also a difference between the 2 intoxicated groups (p<0.05; Bonferroni) indicating a significant improvement in motor function with GCP II inhibitor treatment.

On the fifth day of pyridoxine treatment, there was a significant difference between the three groups in the distance travelled in an open field [F(2, 27) = 24.79; p<0.05; one way ANOVA] (Figure 3d). The pyridoxine/vehicle treated rats had significantly decreased distance travelled compared to the control rats (p<0.05; Newman-Keul). While there was a difference between the pyridoxine/2-MPPA group and the controls (p<0.05; Newman-Keul), there was also a difference between the 2 intoxicated groups (p<0.05; Newman-Keul) indicating a significant improvement in motor function with GCP II inhibition.

### Thermal sensitivity deficits were improved by GCP II inhibition

**Figure 4:**
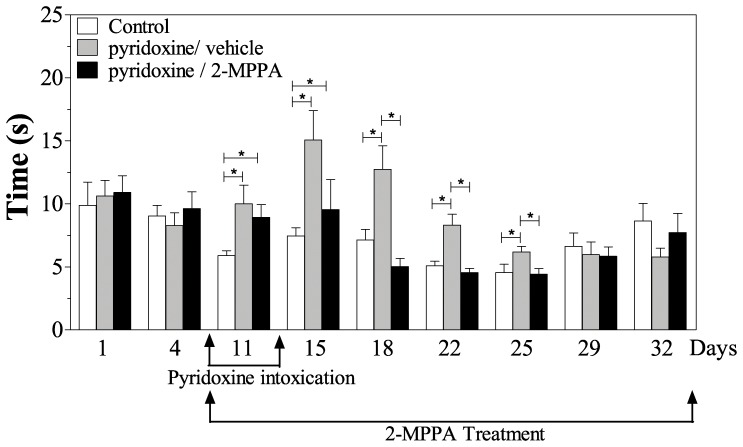
GCP II inhibition improves hot plate reaction times. Pyridoxine intoxication increased reaction time on the hot plate, which was improved by GCP II on days 18–25. By day 29, both of the pyridoxine treated groups had recovered to control levels. N = 10/group. Data = mean ± SEM. * = p<0.05.

Reaction time on the hot plate was normalized in the GCP II inhibitor treated pyridoxine-induced neuropathy rats (Figure 4). There was a significant difference between the 3 groups once the intoxication began [F(2, 24 = 5.78, p<0.05; repeated measures ANOVA] with the pyridoxine/vehicle treated rats being significantly different to both the controls and the pyridoxine/2-MPPA group. An increase in the latency was seen on day 11 and 15 for the 2 pyridoxine treated groups (p<0.05, Fisher’s PLSD). However, this increase was only significant for the pyridoxine/vehicle group from day 18 to day 25 (p<0.05, Fisher’s PLSD). The GCPII inhibitor treated animals displayed no significant difference in the latency versus the control group throughout this period (p>0.05, Fisher’s PLSD) indicating that 2-MPPA was improving heat sensitivity. There was no difference between any groups on days 29 and 32.

### Sensory function deficits were improved by GCP II inhibition

**Figure 5:**
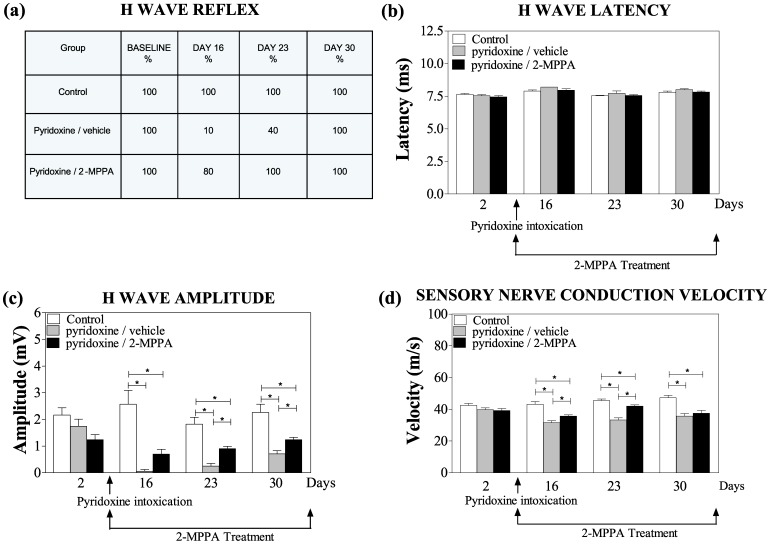
Electromyographical parameters of sensory reflexes were improved by GCP II inhibition. (a) This table represents the percentage of rats that presented with a H wave throughout the study. The H wave reflex was severely attenuated in only the pyridoxine-induced vehicle treated neuropathic rats as indicated by a reduction in the percentage of these rats presenting with H-waves. (b) There was no difference in H wave latency across the entire experiment. (c) On days 16, 23 and 30 the control group had significantly higher H wave amplitudes than pyridoxine treated groups. However, on days 23 and 30, the H wave of the pyridoxine/vehicle group had a smaller amplitude than the pyridoxine/2-MPPA indicating that the 2-MPPA treatment reduced the deficiencies in the H reflex. (d) On days 16 and 23 the pyridoxine/2-MPPA group showed a significant difference with the pyridoxine/vehicle group in sensory nerve conduction velocity indicating a significant improvement with GCP II inhibition. N = 10/group. Data = mean ± SEM. * = p<0.05.

The results of electrophysiological recordings were consistent with pyridoxine-induced toxicity to sensory function. During the baseline week, all the animals showed a reflex H wave. The percentage of rats with H wave reflexes was severely attenuated in pyridoxine-induced vehicle treated neuropathic rats (Figure 5a). On day 16, H waves could only be recorded in one of the ten animals treated with pyridoxine/vehicle versus 80% of animals in the pyridoxine/2-MPPA group (p<0.05; Kruskal-Wallis test). Moreover, on day 23, a H wave was evident in all animals of the pyridoxine/2-MPPA group versus 40% in the pyridoxine/vehicle treated group and by day 30 all animals showed a reflex H wave. The H wave latency showed no difference between the groups across the entire study (figure 5b). However, after the pyridoxine treatment, there was a significant difference in H wave amplitude [F(2, 24 = 38.81, p<0.05; repeated measures ANOVA] with all 3 groups significantly different to each other. On days 16, 23 and 30 the control group had significantly higher H wave amplitudes than both intoxicated groups. However, on days 23 and 30, the H wave of the pyridoxine/vehicle group had a significantly smaller amplitude than the pyridoxine/2-MPPA (p<0.05; Fisher’s PLSD) (Figure 5c) indicating that 2-MPPA rescued the deficiencies in the H reflex. Consistent with previous reports [27], [29] we did not find a difference in the amplitude or latency of the motor M wave (data not shown).

The pyridoxine-induced SNCV deficits were also normalized in GCP II inhibitor treated rats (Figure 5d). A significant difference in SNCV between the 3 groups throughout the study was observed [F(2, 24) = 22.92, p<0.05; repeated measures ANOVA]. A significant decrease was seen following pyridoxine intoxication compared to the control group. A significant difference in the velocity was also seen between the 2 pyridoxine groups [F(1, 17) = 7.75, p<0.05; repeated measures ANOVA]. On days 16 and 23 the pyridoxine/2-MPPA group showed a significant difference from the pyridoxine/vehicle group (p<0.05; Fishers PSLD). Even though the pyridoxine/2-MPPA group was also significantly different to the control group on these days (p<0.05; Fishers PSLD), it still represented a significant improvement compared to the pyridoxine/vehicle group. These data show that GCP II inhibition can significantly improve the electrophysiological impairment caused by high doses of pyridoxine.

### GCP II inhibition prevents neurodegenerative effects

**Figure 6:**
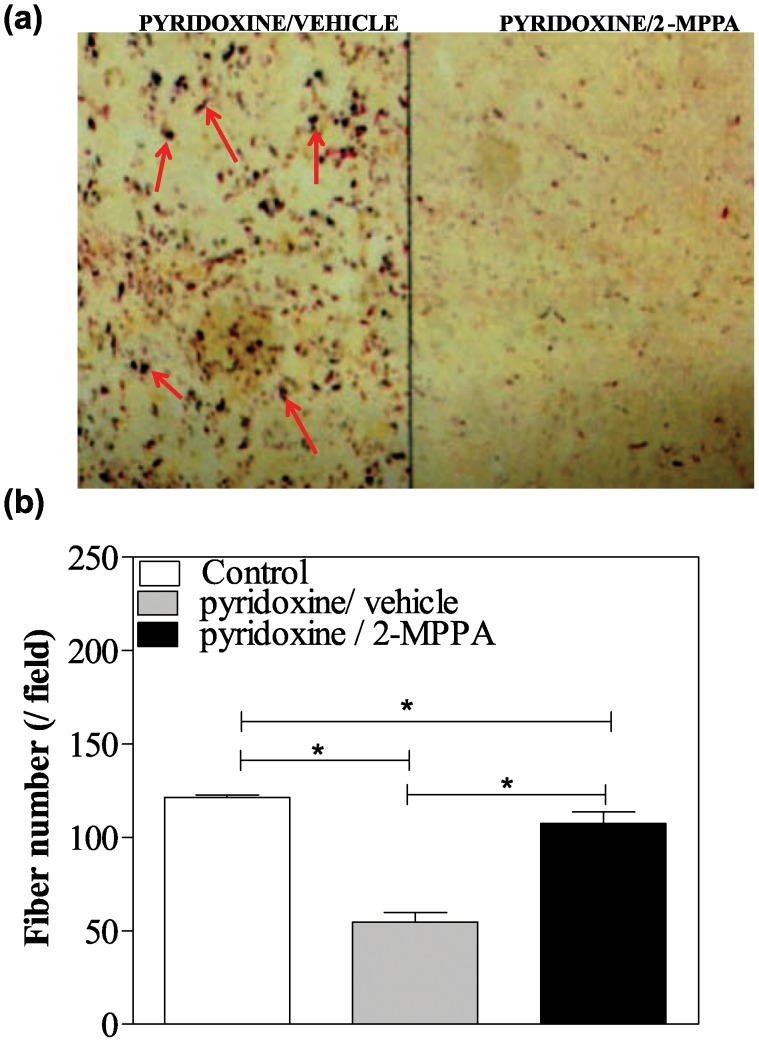
GCP II inhibition protected pyridoxine induced sensory fibers loss in the spinal cord and DRGs. (a) Representative silver-stained photomicrograph of a lumbar spinal cord section from a pyridoxine/vehicle rat and a pyridoxine/2-MPPA rat indicating a clear difference between the groups. There were less large-caliber argyrophillic axonal profiles (red arrows) in rats treated with 2-MPPA. (b) Pyridoxine treatment decreased fiber number in the DRGs, which was also partially prevented by GCPII inhibitor treatment. N = 3/group. Data = mean ± SEM. * = p<0.05.

After 14 days of recovery from the pyridoxine intoxication, the integrity of the central projections of primary proprioceptive afferents of the spinal cord was assessed using a silver stain for degenerating fibers. Figure 6a shows a representative spinal cord section through a lumbar segment from a pyridoxine/vehicle versus pyridoxine/2-MPPA treated rat. Although many large-caliber degenerating argyrophillic axonal profiles were present in the dorsal columns of the pyridoxine-intoxicated rats, they were less prominent in rats treated with 2-MPPA though systematic quantification was not performed.

Comparison of the mean number of sensory fibers in the DRG revealed a significant difference between the 3 groups [F(2, 158) = 167.5; p<0.05; one way ANOVA] (Figure 6b). The average number of fibers per field was 121.35±1.35 in the control group, 55.21±5.11 in the pyridoxine/vehicle and 106.40±6.32 in the pyridoxine/2-MPPA rats. The pyridoxine/vehicle rats had significantly reduced fiber numbers compared to the control group (p<0.05; Bonferroni). While there was still a difference between the pyridoxine/2-MPPA group and the control group (p<0.05; Bonferroni), there was also a difference between the 2 intoxicated groups (p<0.05; Bonferroni) indicating protection from fiber loss with 2-MPPA.

## Discussion

The present study confirms previous reports demonstrating that pyridoxine intoxication in rats induces a severe sensory neuropathy evidenced by behavioral deficits, EMG alterations and pathological changes. Specifically, significant deficits in weight, walking, rotarod and foot fault performance, open field locomotion, and thermal sensitivity were observed. There were also disrupted EMG parameters such as H wave frequency and amplitude and reduced SNCV. Morphological markers of degeneration in the spinal cord and diminished numbers of DRG sensory nerve fibers were also apparent. We report, for the first time, that co-administration of the orally bioavailable GCP II inhibitor, 2-MPPA, protects against the majority of pyridoxine-induced deficits associated with this preclinical model.

Excessive extracellular glutamate is known to cause neurotoxicity [4]. There are several enzymes that can be targeted to reduce excess glutamate levels including inhibition of GCP II. GCP II is a membrane-bound enzyme that cleaves the neuropeptide N-acetylaspartyglutamate (NAAG) into N-acetylaspartate (NAA) and glutamate [1]. NAAG is one of the most abundant peptide transmitters in the brain and is a type 3 metabotropic glutamate receptor (mGluR3) agonist [2], [3]. GCP II is abundantly present in the spinal cord and DRG [12]. GCP II localization has been reported in the satellite cell compartment of DRG’s and in Schwann cells of the sciatic nerve [35]. The mechanism of how GCP II inhibition is capable of rescuing effects of peripheral neuropathy is thought to be through the reduction in glutamate-induced excitotoxicity and increase in NAAG levels, which has also been shown to be neuroprotective [12], [36]. NAAG is a potent agonist of the mGluR3 receptors, whose activation prevents glutamate release and leads to the release of transforming growth factor β (TGFβ), both of which contribute to neuroprotection [2], [12], [37], [38]. In cell culture models of diabetic- and HIV-induced neurodegeneration, mGluR3 receptor activation has been shown to be required for the neuroprotective effect of NAAG and GCP II [39], [40]. To further evaluate the contribution of this mechanism of action of 2-MPPA against pyridoxine neurotoxicity, future studies are planned to perform the pyridoxine –induced neurotoxicity in mGluR3 knock-out mice and to co-administer 2-MPPA with a specific mGluR3 antagonists as described in earlier studies [39], [40]. NAAG also partially antagonizes NMDA receptors which could also contribute to its neuroprotective effects [41] although this observation is controversial [42], [43]. It has previously been shown that GCP II inhibition is capable of protecting against other models of peripheral neuropathy, including diabetic and chemotherapy-induced [4], [12], [13], [39], [44]. In further support, GCP II knock-out mice were shown to be significantly less susceptible to the motor, electrophysiological and pathological detrimental effects of sciatic nerve crush when directly compared to wild type mice [36].

GCP II inhibition may have benefits over existing glutamatergic therapies such as potent postsynaptic glutamatergic therapies which have been fraught with side-effects [45]. 2-MPPA has, in fact, been administered to human volunteers and was well tolerated with no reports of adverse CNS effects [18]. Also, since GCP II inhibitors do not act directly at a glutamatergic receptor, but rather reduce extracellular glutamate levels, they have the potential to influence several types of glutamatergic receptors rather than specifically at a single subtype. Finally, GCP II inhibitors are also beneficial over other glutamatergic drugs as their effects seem to be specific to conditions where increased glutamate transmission occurs rather than at basal glutamate levels [11], [36], [46], [47] and thus may be more tolerated with less side effects as other therapeutics.

In the current study, several of the pyridoxine-induced deficits were improved by GCP II inhibition. For example, severe deficits in the number and amplitude of H-wave reflexes observed after pyridoxine treatment were partially prevented with 2-MPPA treatment. The H-wave deficits were accompanied by reductions in SNCV, which was also partially prevented by GCPII inhibition. H-wave reflexes provide valuable information about functionally important features of peripheral sensory nerves and their synaptic connections to the central nervous system [48]. Consistent with previous reports that the pyridoxine intoxication selectively targets sensory nerve function [27], [29], we did not find a difference in the amplitude or latency of the motor M wave.

Spinal cord and DRG morphological analysis also confirmed neuroprotection by GCP II inhibition. Qualitative assessment of sections found many large-caliber degenerating argyrophillic axonal profiles were present in the dorsal columns of the pyridoxine-intoxicated rats but were less prominent in rats treated with 2-MPPA. These profiles are thought to be the ascending collaterals of afferents that degenerate as a consequence of a high dose pyridoxine administration [26]. The almost complete absence of the H-wave reflex after pyridoxine treatment versus the significantly less severe loss in the GCPII treated animals provides credence to these morphological findings. In addition, this is also supported by the increased number of sensory fibers in the lumbar DRG from rats treated with 2-MPPA compared to vehicle treated pyridoxine rats, as H-wave reflex also involve the DRG sensory neurons.

The deficiencies in electrophysiological measurements and morphological parameters were accompanied by functional impairments in locomotor abilities, coordination, and in thermal sensitivity. Consistent with improvements in the H-wave reflex, SNCV and morphological degeneration, the majority of these behavioral effects due to pyridoxine treatment were also prevented or rescued by GCP II inhibition.

There is a possibility that protective effects demonstrated on the hot plate test could be as a result of analgesic effects of 2-MPPA since the hotplate was tested 1 hour after 2-MPPA administration and analgesic effects of 2-MPPA have been observed after this time period in other studies [22]. However, the morphological and electrophysiological improvements shown in this study argue against this as the sole mechanism but rather suggest functional neuroprotective changes associated with chronic 2-MPPA treatment. There is also a possibility that GCP II could be interfering with the neurotoxic action of pyridoxine, though some of the positive effects of 2-MPPA we observed appear weeks after pyridoxine withdrawal making this conclusion less probable. In addition, GCP II knock-out mice were shown to be significantly less susceptible to the effects of sciatic nerve crush and ischemic brain injury [36] suggesting GCP II inhibition may not simply be blocking pyridoxine toxicity. Future studies should include pyridoxine toxicity in GCP II knock-out mice to confirm this. It would also be interesting to evaluate if selective mGluR3 agonists could also provide neuronal protection in this neuropathy model.

### Conclusion

In conclusion, we have demonstrated, for the first time, that the orally bioavailable GCPII inhibitor, 2-MPPA, protects against the severe neuronal injury caused by the pyridoxine treatment. Although we have not demonstrated a curative effect in the present study, attempts by other groups to completely prevent this type of neuropathy have also been unsuccessful [26], [27], [49], [50], [51]. In conjunction with the previously described beneficial effects of GCPII inhibition in diabetic and chemotherapy-induced neuropathy [12], [13], the data support the possible broad therapeutic utility of GCPII inhibition in alleviating the symptoms of peripheral nervous system injuries.
